# Preoperative GNRI and surgical site infection risk after total joint arthroplasty: A systematic review and meta-analysis

**DOI:** 10.17305/bb.2026.13414

**Published:** 2026-02-27

**Authors:** Mingchang Du, Tiejun Liu, Ye Liu, Jie Guo, Xu Ma, Liangquan Zhai, Jun Li, Tiejun Wang, Hong Gao, Xun Fu

**Affiliations:** 1 Orthopedics Department, The Orthopedic Hospital of Shenyang, Shenyang, China; 2 Department of Imaging, Tacheng People’s Hospital of Xinjiang Uygur Autonomous Region, Tacheng, China; 3 Department of Imaging, The Orthopedic Hospital of Shenyang, Shenyang, China

**Keywords:** Total joint arthroplasty, malnutrition, geriatric nutritional risk index, surgical site infection, periprosthetic joint infection

## Abstract

Surgical site infections (SSIs), including periprosthetic joint infections (PJIs), represent significant complications following total joint arthroplasty (TJA). The geriatric nutritional risk index (GNRI) serves as an objective measure of nutritional status; however, its predictive value for postoperative infections in TJA patients remains ambiguous. To address this issue, a meta-analysis was conducted alongside a systematic search of PubMed, Embase, and Web of Science. Observational studies that assessed the relationship between preoperative GNRI and postoperative SSI or PJI following TJA were included in the analysis. Odds ratios (ORs) with 95% confidence intervals (CIs) were synthesized using random-effects models to account for heterogeneity. Seven retrospective cohort studies comprising 221,810 patients were analyzed. The results indicated that a low GNRI, which suggests malnutrition, is significantly associated with an increased risk of SSI after TJA (OR = 1.59; 95% CI 1.21–2.08; I^2^ ═ 81%; *P <* 0.001). This association persisted across both primary and revision procedures (OR = 1.52 vs. 1.65; *P ═* 0.76) and within 30-day and 90-day follow-ups (OR = 1.58 vs. 1.56; *P ═* 0.98). A more pronounced correlation was found in patients with severe malnutrition (GNRI < 92) compared to those with moderate risk (GNRI: 92–98; OR = 2.18 vs. 1.25; *P <* 0.001). Although a similar trend was observed for PJI (OR = 2.37; 95% CI 0.73–7.72; *P ═* 0.15), this finding was based on only two studies (three datasets) and remains uncertain. Evidence regarding shoulder arthroplasty was limited; however, existing data suggested trends similar to those seen in hip and knee arthroplasty. In conclusion, preoperative malnutrition, as indicated by a low GNRI, may be associated with an elevated risk of SSIs following TJA.

## Introduction

Total joint arthroplasty (TJA), encompassing total hip arthroplasty (THA), total knee arthroplasty (TKA), and total shoulder arthroplasty (TSA), ranks among the most successful and frequently performed orthopedic procedures globally [1, 2]. With the aging global population and the expansion of surgical indications, the annual volume of TJA is rapidly increasing, surpassing 2 million procedures worldwide and projected to double within the next decade [3]. TJA significantly enhances pain relief, functional capacity, and quality of life for patients suffering from end-stage joint diseases, such as osteoarthritis and rheumatoid arthritis [4, 5]. Nevertheless, despite advancements in surgical techniques, aseptic measures, and perioperative management, postoperative complications persist, with surgical site infection (SSI), including periprosthetic joint infection (PJI), being among the most severe [6, 7]. SSI leads to prolonged hospitalization, delayed rehabilitation, and considerable healthcare costs, and may necessitate revision surgery, implant removal, or even result in permanent disability or death [8, 9]. Thus, identifying risk factors for SSI is critical for effective prevention and improved outcomes following TJA.

The geriatric nutritional risk index (GNRI) serves as a straightforward, objective measure of nutritional status, initially developed to assess malnutrition-related risks in older adults [10, 11]. It is computed using serum albumin concentration and the ratio of actual to ideal body weight, represented as: GNRI ═ [1.489 × serum albumin (g/L)] + [41.7 × (body weight/ideal body weight)] [10]. Lower GNRI values signify poorer nutritional status. Although originally applied to elderly populations, GNRI has gained traction in various clinical contexts, including cardiovascular [12, 13], gastrointestinal [14], and orthopedic surgeries [15], as a prognostic marker for postoperative complications. Malnutrition, as indicated by low GNRI, may compromise immune function, delay wound healing, and heighten infection risk through mechanisms involving diminished protein synthesis, impaired collagen deposition, and weakened inflammatory responses [16, 17]. Although several recent studies have investigated the correlation between preoperative GNRI and postoperative infections following TJA, their findings have been inconsistent, potentially due to variations in surgical types, infection definitions, and GNRI cutoffs [18–24]. To address these discrepancies and provide quantitative evidence, this meta-analysis aims to systematically evaluate the association between preoperative GNRI and the risk of SSI, including PJI, after TJA.

## Materials and methods

This meta-analysis adhered to the Preferred Reporting Items for Systematic Reviews and Meta-Analyses (PRISMA) 2020 guidelines [25] and the Cochrane Handbook for Systematic Reviews and Meta-Analyses [26] for protocol development, data extraction, statistical analysis, and results reporting. The study protocol was registered in International Prospective Register of Systematic Reviews (PROSPERO) under ID CRD420251173211, with no deviations from the registered eligibility criteria.

### Literature search

Studies were identified through a comprehensive search in PubMed, Embase, and Web of Science, utilizing a wide array of search terms, including: (1) “geriatric nutritional risk index” OR “GNRI” OR “malnutrition” OR “nutritional indices”; (2) “total hip arthroplasty” OR “total knee arthroplasty” OR “total shoulder arthroplasty” OR “total joint arthroplasty” OR “total hip replacement” OR “total knee replacement” OR “total shoulder replacement” OR “total joint replacement” OR “THA” OR “TKA” OR “TJA” OR “joint replacement” OR “arthroplasty.” The search was limited to human studies and full-length articles published in English in peer-reviewed journals. Additionally, references from relevant original and review articles were manually screened for additional eligible studies. The search encompassed database inception to September 25, 2025. The complete search strategy for each database is detailed in Supplemental File 1.

### Inclusion and exclusion criteria

The eligibility criteria for studies were defined based on the Population, Intervention, Comparison, Outcomes, Study design (PICOS) framework:

- **Population (P)**: Adult patients (≥ 18 years) undergoing primary or revision TJA, including THA, TKA, or TSA.
- **Intervention (I)**: Patients exhibiting nutritional risk indicated by a low GNRI before surgery, as defined by cutoffs in each included study.
- **Comparison (C)**: Patients without nutritional risk, characterized by a high GNRI, consistent with the cutoffs defined in each study.
- **Outcomes (O)**: The primary outcome was the incidence of overall SSI, while the secondary outcome was the incidence of PJI, as defined by the original studies, compared between patients with low vs normal GNRI prior to surgery.
- **Study design (S)**: Studies with longitudinal follow-up were included, such as prospective or retrospective cohort studies, nested case-control studies, or post-hoc analyses of clinical trials.

Studies were excluded if they met any of the following criteria: (1) not limited to patients receiving TJA (e.g., fracture fixation, spinal, or other orthopedic surgeries); (2) did not report GNRI or provide sufficient data to estimate the association between GNRI and SSI; (3) included pediatric patients or animal studies; (4) were case reports, reviews, editorials, or duplicate publications from the same cohort; or (5) lacked clear definitions of postoperative infection outcomes or reported only non-infectious complications. In cases of population overlap, the study with the largest sample size was selected for inclusion in the meta-analysis. To assess potential population overlap among studies utilizing large administrative databases, we compared study periods, procedure types, and inclusion criteria. Studies with overlapping patient populations were excluded to prevent duplication and inflation of precision.

### Study quality assessment and data extraction

Two authors (M.D. and T.L.) independently conducted the literature search, study selection, quality assessment, and data extraction, resolving discrepancies through discussion with the corresponding author. Study quality was evaluated using the Newcastle–Ottawa Scale (NOS) [27], which assesses selection, confounding control, and outcome measurement, with scores ranging from 1 to 9, where 9 indicates the highest quality. Studies with NOS scores of 7 or above were considered high quality. Data extracted for analysis included study characteristics (author, year, study design, and country), types of TJA (primary or revision, and replaced joint locations), patient characteristics (sample size, mean age, and sex distribution), exposure characteristics (GNRI measurement timing, cutoffs for defining low GNRI, and number of patients at risk of malnutrition according to GNRI), follow-up durations, reported outcomes, incidence of overall SSI, and variables adjusted in estimating the relationship between GNRI and SSI following TJA.

**Figure 1.**
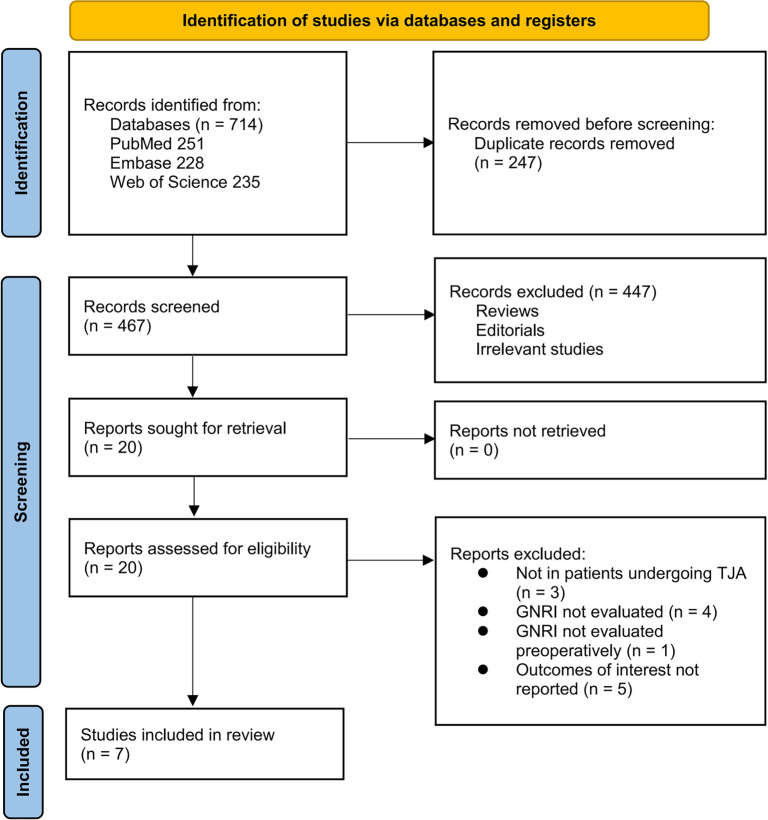
**Flowchart of database search and study inclusion**.

### Statistical analyses

The association between GNRI and the risk of SSI and PJI after TJA was presented as odds ratios (ORs) with corresponding 95% confidence intervals (CIs), compared between patients with low vs normal GNRI prior to surgery. In cases where studies reported multiple models, we extracted the most fully adjusted OR to minimize confounding and ensure comparability across studies. Unadjusted estimates were utilized only when no adjusted OR was available. Consequently, the primary pooled analysis was based on the most adjusted effect from each study. For studies that reported various levels of malnutrition risk according to GNRI categories (e.g., moderate and severe risk compared to a common normal-GNRI reference), these contrasts were extracted and included as separate datasets in the meta-analysis. This methodology preserves clinically relevant information regarding GNRI severity and facilitates the assessment of potential dose–response relationships. As extracted effect estimates were regression-derived ORs accompanied by their respective standard errors, they were treated as independent comparisons for statistical pooling. However, it is acknowledged that contrasts originating from the same cohort may introduce within-study correlation and are not fully independent at the population level. Given the limited number of eligible studies and the consistency of effect directions across GNRI categories, this approach was deemed appropriate, with explicit acknowledgment of its potential impact on precision. For studies providing only raw event counts, unadjusted ORs were calculated, applying a continuity correction of 0.5 to all four cells if any cell contained zero events. All extracted or derived effect sizes were converted to ORs to ensure consistency across analyses. ORs and their standard errors were computed from 95% CIs or *P* values and log-transformed to stabilize variance and normalize distribution [26]. Heterogeneity was assessed using the Cochrane *Q* test and I^2^ statistics [28], with I^2^ values < 25%, 25%–75%, and > 75% indicating mild, moderate, and substantial heterogeneity, respectively. A random-effects model was employed to synthesize results while accounting for variability across studies [26]. Additionally, τ^2^ was reported, and a 95% prediction interval (PI) was calculated for the primary random-effects model [26]. Sensitivity analyses were conducted using a leave-one-dataset-out approach to determine whether any single effect estimate disproportionately influenced the pooled result [29]. Furthermore, predefined subgroup analyses were performed to evaluate study characteristics on the results, such as type of TJA (primary vs. revision), location of replaced joints (THA/TKA vs. TSA), severity of malnutrition risk indicated by GNRI category, follow-up durations, and analytic models (univariate vs. multivariate analyses). For subgroup analyses based on categorical factors (procedure type, joint type, GNRI categories, follow-up duration, and analytic model), we utilized classifications reported in the original studies. For subgroup analyses involving continuous study-level variables without established cutoffs, we dichotomized the variable based on its median value across studies. Publication bias was assessed through funnel plots, visual asymmetry inspection, and Egger’s regression test [30]. A *P* value < 0.05 was considered statistically significant. Statistical analyses were performed using RevMan (Version 5.3; Cochrane Collaboration, Oxford, UK) and Stata software (version 17.0; Stata Corporation, College Station, TX, USA).

## Results

### Study identification

Figure 1 illustrates the study selection process. Initially, 714 records were identified across three databases, resulting in the removal of 247 duplicates. Following title and abstract screening, 447 articles were excluded for not meeting the criteria for meta-analysis. The full texts of the remaining 20 studies were independently reviewed by two authors, leading to the exclusion of 13 studies for reasons detailed in Figure 1. Ultimately, seven studies were included in the quantitative analysis [18–24].

### Overview of study characteristics

Table 1 summarizes the characteristics of the seven retrospective cohort studies included in this meta-analysis, all conducted in the United States between 2022 and 2025 [18–24]. These studies assessed the association between the GNRI and postoperative SSIs or PJI following TJA. A total of 221,810 patients were included, with six studies focusing on older adults aged ≥ 65 years [18–22, 24] and one study involving adult patients undergoing TJA [23]. Three studies included patients undergoing primary TJA [18, 21, 23], while the remaining four studies focused on revision TJA [19, 20, 22, 24]. With regard to the joints being replaced, five studies included patients receiving TKA or THA [18–20, 23, 24], whereas the other two studies involved patients receiving TSA [21, 22]. The GNRI was measured preoperatively, with commonly applied cutoff values classified as normal (> 98), moderate (92–98), and severe (< 92) nutritional risk. One study used a modified GNRI (mGNRI) and identified an optimal threshold of 92.8 for predicting postoperative complications [23]. This value is close to the conventional cutoff of 92 for severe nutritional risk; thus, we harmonized it to the GNRI < 92 category. To ensure robustness, we also performed a sensitivity analysis excluding this dataset. Consequently, 39,170 patients (17.7%) were identified as being at malnutrition risk prior to surgery based on the GNRI. Follow-up periods for detecting SSI or PJI were 30 days in five studies [18, 20–22, 24] and 90 days in two studies [19, 23]. The primary outcome of SSI was reported in all studies [18–24], while the secondary outcome of PJI was reported in two studies [19, 23]. Detailed definitions of SSI/PJI and the time window for each study are summarized in Table S1. Overall, 2,472 patients (1.1%) developed SSI after TJA. Five studies [18, 20, 21, 23, 24] conducted multivariable-adjusted analyses, controlling for potential confounders such as age, sex, body mass index, comorbidities (e.g., diabetes, congestive heart failure, chronic obstructive pulmonary disease), functional status, and American Society of Anesthesiologists classification when estimating the association between GNRI and the risk of SSI after TJA. The remaining two studies [19, 22] reported only univariate analysis data.

### Study quality evaluation

As presented in Table 2, methodological quality was assessed using the NOS. Total NOS scores ranged from 5–9, indicating moderate to high methodological quality among the included studies. Four studies scored 7, reflecting adequate control for key confounders, clear outcome ascertainment, and sufficient cohort follow-up [18, 20, 21, 24]. The study by Hansen et al. [23] achieved the highest score of 9, demonstrating strong representativeness, comprehensive confounder adjustment, and complete follow-up. Two studies scored 5 and 6, primarily due to limited confounder adjustment [19, 22]. Overall, most studies provided robust data with appropriate adjustments for demographic and clinical variables, supporting the reliability of pooled estimates linking preoperative GNRI with postoperative SSI/PJI risk in patients undergoing TJA.

**Table 1:** Characteristics of the included studies

**Study**	**Design**	**Country**	**Surgery type**	**Sample size**	**Mean age (years)**	**Men (%)**	**Timing of GNRI measurement**	**Cutoff of GNRI**	**Number of patients at risk for malnutrition**	**Follow-up duration (days)**	**SSI outcomes reported**	**Number of patients with SSI**	**Variables adjusted**
Fang 2022	RC	USA	Primary THA and TKA	191,087	NR, all ≥65 years	60.3	Preoperative	Normal: >98; Moderate: 92–98; Severe: <92	30,258	30	Overall SSI	1,791	Age, sex, BMI, 5i-mFI, ASA class, smoking, anemia, preoperative dialysis, and body weight loss
Oakley 2023	RC	USA	Revision THA and TKA	531	73.1, all ≥65 years	41.8	Preoperative	Normal: >98; Moderate: 92–98; Severe: <92	67	90	Overall SSI and PJI	50	None
Lung 2024	RC	USA	Revision THA (aseptic)	7,119	NR, all ≥65 years	41.4	Preoperative	Normal: >98; Moderate: 92–98; Severe: <92	2,777	30	Overall SSI	312	Age, sex, BMI, functional status, ASA classification, smoking status, chronic steroid use, CHF, diabetes, COPD, bleeding disorders, and disseminated cancer
Liu 2024a	RC	USA	Primary TSA	11,411	NR, all ≥65 years	40.5	Preoperative	Normal: >98; Moderate: 92–98; Severe: <92	2,365	30	Overall SSI	42	Age, sex, BMI, functional status, ASA classification, smoking status, steroid use, CHF, diabetes, COPD, bleeding disorders, disseminated cancer
Liu 2024b	RC	USA	Revision TSA	678	NR, all ≥65 years	45.3	Preoperative	Normal: >98; Moderate: 92–98; Severe: <92	160	30	Overall SSI	17	None
Liu 2025	RC	USA	Revision TKA	9,409	NR, all ≥65 years	41.7	Preoperative	Normal: >98; Moderate: 92–98; Severe: <92	2,751	30	Overall SSI	216	Age, sex, BMI, functional status, ASA, tobacco use, steroid use, diabetes, hypertension, COPD, bleeding disorders, CHF, disseminated cancer
Hansen 2025	RC	USA	Primary THA and TKA	1,575	66.5	43.5	Preoperative	Severe: <92	792	90	Overall SSI and PJI	44	Age, sex, BMI, CCI, and surgery type

Abbreviations: RC: Retrospective cohort; THA: Total hip arthroplasty; TKA: Total knee arthroplasty; TSA: Total shoulder arthroplasty; SSI: Surgical site infection; PJI: Periprosthetic joint infection; GNRI: Geriatric nutritional risk index; BMI: Body mass index; 5i-mFI: 5-item modified frailty index; ASA: American Society of Anesthesiologists; CHF: Congestive heart failure; COPD: Chronic obstructive pulmonary disease; CCI: Charlson comorbidity index; NR: Not reported;

**Table 2:** Study quality evaluation via the Newcastle-Ottawa Scale

**Study**	**Representativeness of the exposed cohort**	**Selection of the** **non-exposed** **cohort**	**Ascertainment of exposure**	**Outcome not present at baseline**	**Control for age**	**Control for other confounding factors**	**Assessment of outcome**	**Sufficient follow-up duration**	**Adequacy of** **follow-up** **of cohort**	**Total**
Fang 2022	0	1	1	1	1	1	1	0	1	7
Oakley 2023	0	1	1	1	0	0	1	1	1	6
Lung 2024	0	1	1	1	1	1	1	0	1	7
Liu 2024a	0	1	1	1	1	1	1	0	1	7
Liu 2024b	0	1	1	1	0	0	1	0	1	5
Liu 2025	0	1	1	1	1	1	1	0	1	7
Hansen 2025	1	1	1	1	1	1	1	1	1	9

### Meta-analysis results

**Figure 2.**
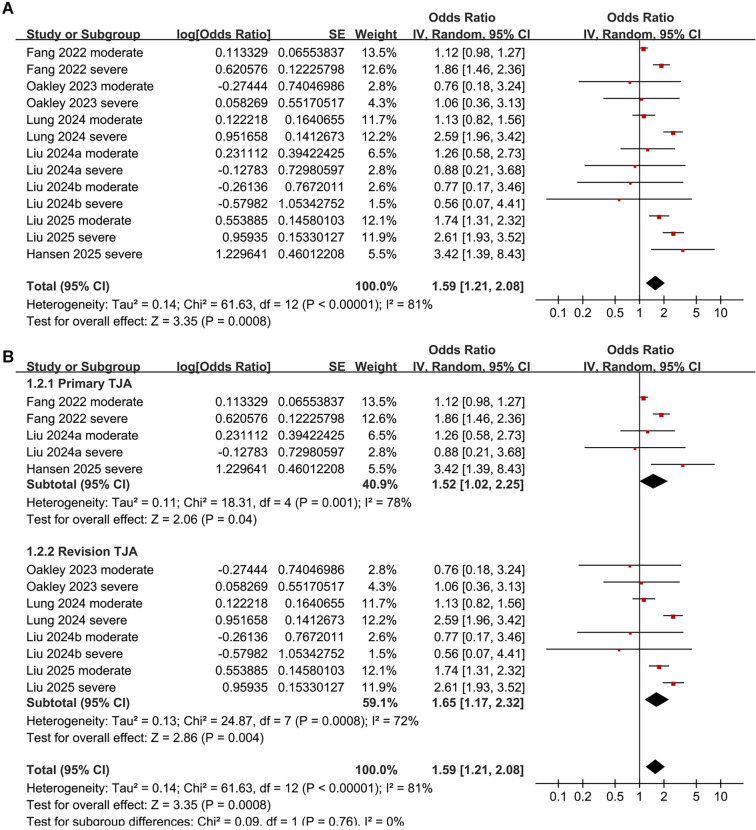
**Forest plots of the association between preoperative nutritional risk assessed by the GNRI and postoperative SSI after TJA**. (A) Overall random-effects meta-analysis pooling 13 GNRI-category datasets from seven retrospective cohort studies; when studies reported GNRI severity strata (e.g., moderate and severe malnutrition risk), each stratum was entered as a separate dataset. Low GNRI (malnutrition risk) was associated with increased SSI risk (OR 1.59, 95% CI 1.21–2.08; I^2^ ═ 81%; τ^2^ ═ 0.14; 95% prediction interval 0.73–3.47). (B) Subgroup analysis by procedure type shows comparable associations in primary and revision TJA (primary: OR 1.52, 95% CI 1.02–2.25; revision: OR 1.65, 95% CI 1.17–2.32; *P* for subgroup difference = 0.76). Abbreviations: CI: Confidence interval; GNRI: Geriatric nutritional risk index; OR: Odds ratio; PI: Prediction interval; SSI: Surgical site infection; TJA: Total joint arthroplasty.

**Figure 3.**
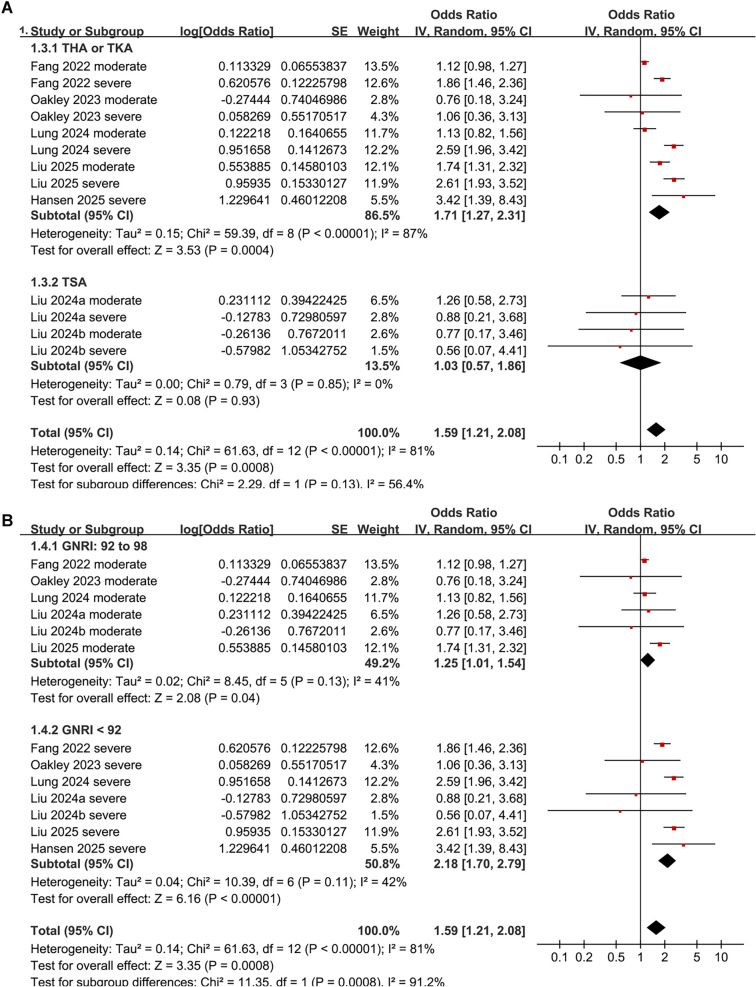
**Forest plots of prespecified subgroup analyses evaluating the association between preoperative malnutrition assessed by the GNRI and postoperative SSI after TJA.** (A) Subgroup analysis by joint site shows that low GNRI was associated with higher SSI risk after hip or knee arthroplasty (THA/TKA: OR 1.71, 95% CI 1.27–2.31), whereas no clear association was observed in the shoulder arthroplasty subgroup (TSA: OR 1.03, 95% CI 0.57–1.86); the between-subgroup difference was not statistically significant (*P ═* 0.13). (B) Subgroup analysis by GNRI-defined malnutrition severity demonstrates a graded association, with a smaller effect in moderate nutritional risk (GNRI 92–98: OR 1.25, 95% CI 1.01–1.54) and a stronger effect in severe nutritional risk (GNRI < 92: OR 2.18, 95% CI 1.70–2.79); the subgroup difference was significant (*P ═* 0.0008). Random-effects models were used throughout. Abbreviations: CI: Confidence interval; GNRI: Geriatric nutritional risk index; OR: Odds ratio; SSI: Surgical site infection; THA: Total hip arthroplasty; TKA: Total knee arthroplasty; TSA: Total shoulder arthroplasty; TJA: Total joint arthroplasty.

**Figure 4.**
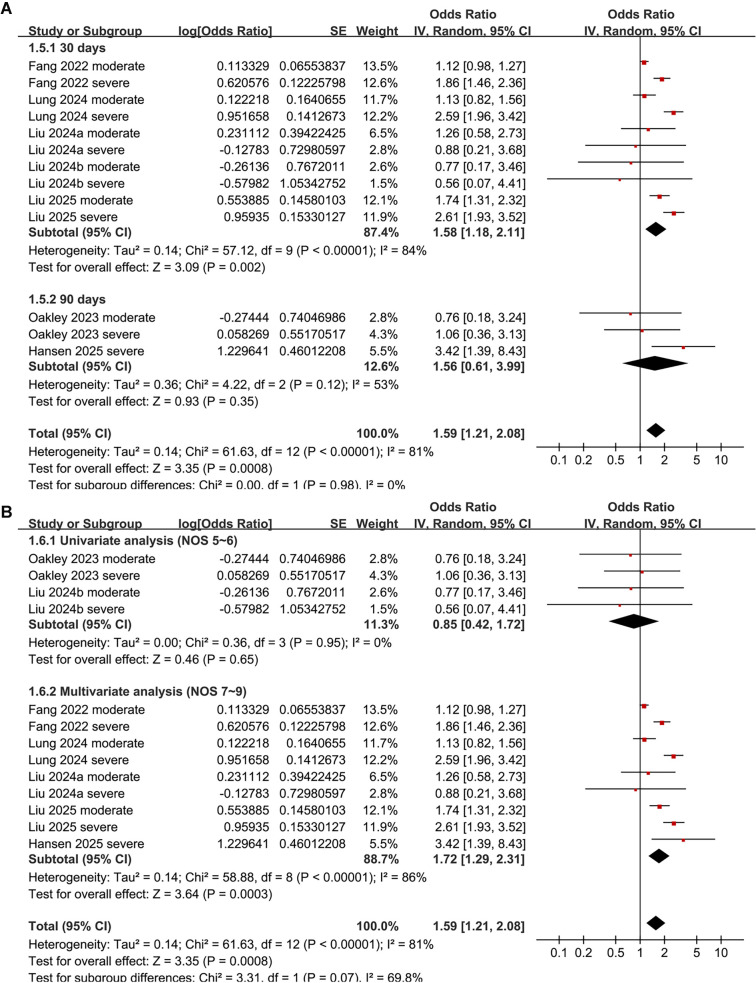
**Forest plots of prespecified subgroup analyses evaluating the robustness of the association between preoperative malnutrition, as indicated by a low GNRI, and the incidence of postoperative SSI following TJA.** (A) Stratified by follow-up window, the association was comparable in studies using 30-day surveillance (OR 1.58, 95% CI 1.18–2.11) and those using 90-day surveillance (OR 1.56, 95% CI 0.61–3.99), with no evidence of a between-subgroup difference (*P ═* 0.98). (B) Stratified by analytic approach, the pooled association was driven primarily by studies reporting multivariable-adjusted estimates (OR 1.72, 95% CI 1.29–2.31), whereas studies reporting univariate estimates showed no clear association (OR 0.85, 95% CI 0.42–1.72); the between-subgroup difference did not reach statistical significance (*P ═* 0.07). Studies contributing multivariable estimates generally had higher NOS scores for confounding control (NOS 7–9 vs. 5–6), and this panel therefore also serves as a quality-oriented sensitivity analysis. Random-effects models were used throughout. Abbreviations: CI: Confidence interval; GNRI: Geriatric nutritional risk index; NOS: Newcastle–Ottawa Scale; OR: Odds ratio; SSI: Surgical site infection; TJA: Total joint arthroplasty.

Six studies [18–22, 24] provided data according to the severity of malnutrition risk by GNRI categories, which were included in the meta-analysis independently. Pooled results from 13 datasets across the seven studies [18–24] indicated that malnutrition, as evidenced by a low GNRI, was associated with a higher risk of SSI after TJA (OR: 1.59, 95% CI: 1.21–2.08, *P*< 0.001; I^2^ ═ 81%; Figure 2A). The between-study variance was τ^2^ ═ 0.14. The 95% PI for the primary random-effects model ranged from 0.73–3.47, suggesting that while the pooled effect favored an increased infection risk with low GNRI, the true effect in future similar settings may range from no association to a substantially elevated risk. Sensitivity analyses, excluding one dataset at a time, yielded consistent results (OR: 1.49–1.73, *P* all< 0.05). Excluding the study employing mGNRI [23] produced similar outcomes (OR: 1.52, 95% CI: 1.16–2.00, *P*= 0.003; I^2^ ═ 81%). Further subgroup analyses indicated consistent results in patients undergoing primary and revision TJA (OR: 1.52 vs. 1.65, *P* for subgroup difference = 0.76; Figure 2B). Notably, a lower GNRI was associated with increased SSI risk following THA or TKA, but not in patients undergoing TSA (OR: 1.71 vs. 1.03), although the difference between subgroups was not statistically significant (*P* for subgroup difference = 0.13; Figure 3A). Furthermore, the risk of postoperative SSI was more pronounced in patients with a preoperative GNRI < 92 compared to those with a preoperative GNRI between 92 and 98 (OR: 2.18 vs. 1.25, *P* for subgroup difference < 0.001; Figure 3B). The association remained similar for studies with follow-up durations of 30 days and 90 days (OR: 1.58 vs. 1.56, *P* for subgroup difference = 0.98; Figure 4A). Finally, results were primarily influenced by studies employing multivariable analyses rather than univariable analyses (OR: 1.72 vs. 0.85), although the between-subgroup difference was not significant (*P* for subgroup difference = 0.07; Figure 4B).

**Figure 5.**
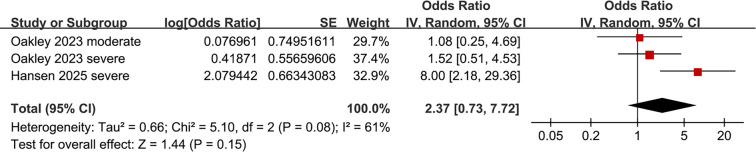
**Forest plot of an exploratory random-effects meta-analysis of the association between low preoperative GNRI and PJI after TJA.** Only two studies (three datasets) contributed data, and the pooled association was not statistically significant (OR 2.37, 95% CI 0.73–7.72; *P ═* 0.15; I^2^ ═ 61%). Given the limited evidence and wide CIs, results should be interpreted cautiously and study-level estimates may be more informative than the pooled effect. Abbreviations: CI: Confidence interval; GNRI: Geriatric nutritional risk index; OR: Odds ratio; PJI: Periprosthetic joint infection; TJA: Total joint arthroplasty.

An exploratory meta-analysis involving three datasets from two studies [19, 23] did not reveal a statistically significant association between low GNRI and the risk of PJI after TJA (OR: 2.37, 95% CI: 0.73–7.72, *P*= 0.15; I^2^ ═ 61%; Figure 5). The confidence interval was wide, reflecting substantial imprecision due to the limited number of contributing studies and events. In this context, individual study-level estimates and their associated uncertainties provide more informative insights than the exact value of pooled ORs, precluding any definitive conclusions about PJI risk at this stage.

### Publication bias

**Figure 6.**
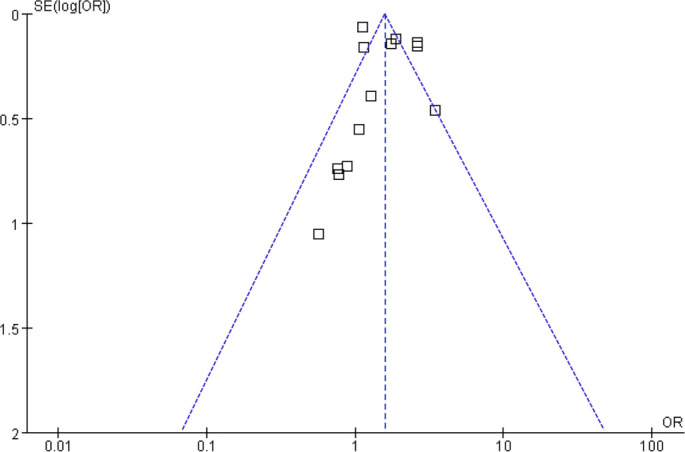
**Funnel plot for assessing potential publication bias in meta-analyses of the association between GNRI and the risk of SSI following TJA.** No clear asymmetry was observed, and Egger’s test was not significant (*P ═* 0.31); however, findings are exploratory given heterogeneity and the limited evidence base. Abbreviations: GNRI: Geriatric nutritional risk index; SSI: Surgical site infection; TJA: Total joint arthroplasty.

Figure 6 presents funnel plots assessing publication bias in the meta-analysis of the association between low GNRI and the risk of SSI after TJA. The funnel plots did not exhibit clear visual asymmetry upon inspection, and Egger’s regression test did not indicate statistically significant small-study effects (*P ═* 0.31). However, due to the limited number of included studies, substantial heterogeneity, and the presence of multiple datasets derived from the same cohorts, these assessments should be regarded as exploratory and interpreted with caution. Assessment of publication bias related to the association between low GNRI and the risk of PJI was not feasible due to the inclusion of only three datasets.

## Discussion

This meta-analysis represents the first quantitative synthesis of evidence regarding the relationship between the GNRI and SSI, including PJI, following TJA. By aggregating data from seven retrospective cohort studies involving over 220,000 patients, this analysis demonstrates that preoperative malnutrition, indicated by a lower GNRI, is significantly associated with an elevated risk of postoperative SSI following TJA. This association persisted across various types of arthroplasty procedures and follow-up durations, particularly among patients classified as severely malnourished (GNRI < 92). While a similar trend was observed for PJI, the association did not achieve statistical significance, likely due to the limited number of included studies and the smaller number of infection events. These findings underscore the growing recognition of nutritional status as a crucial, yet modifiable, determinant of postoperative outcomes in patients undergoing joint replacement surgery.

The observed association between low GNRI and increased SSI risk after TJA may be elucidated through several interrelated biological mechanisms. The GNRI is derived from serum albumin concentration and the ratio of actual to ideal body weight—parameters that collectively reflect both protein reserves and body composition [10]. Serum albumin serves as a key determinant of colloid osmotic pressure and an indicator of systemic inflammatory and nutritional states [31, 32]. Hypoalbuminemia may impair immune function, diminish complement activation, and delay fibroblast proliferation, thereby compromising wound healing and the ability to combat infection [33]. Concurrently, low body weight or muscle mass may signify protein-energy malnutrition, leading to decreased collagen synthesis, impaired angiogenesis, and reduced tissue perfusion[34–36]. These mechanisms collectively undermine the integrity of the surgical wound and heighten susceptibility to bacterial invasion. Consequently, a low GNRI acts as a comprehensive marker encompassing both inflammatory burden and nutritional deficiency, which together predispose patients to postoperative infectious complications [37].

The subgroup and sensitivity analyses further reinforce the robustness and interpretability of the main findings. The similar effect sizes observed in both primary and revision arthroplasty procedures suggest that the adverse impact of poor nutritional status on infection risk remains consistent, irrespective of surgical complexity. Although revision procedures typically carry a higher baseline risk of infection due to longer operative times, greater tissue dissection, and previous scar formation, malnutrition appears to exert a comparable relative influence across both surgical categories [38]. Similarly, the comparable results between the 30-day and 90-day follow-up periods indicate that preoperative GNRI is relevant for predicting both early and subacute postoperative infections. Notably, the stronger association observed in patients with severe malnutrition (GNRI < 92) compared to those with moderate malnutrition (GNRI 92–98) supports a dose–response relationship, underscoring the incremental infection risk associated with progressive nutritional decline. This graded effect provides a clinical rationale for stratifying patients according to GNRI severity to guide perioperative optimization strategies.

The subgroup analysis by surgical site revealed that the association between GNRI and infection risk was significant in studies involving hip or knee arthroplasty, but not in those limited to shoulder arthroplasty. This discrepancy likely reflects differences in both sample size and surgical context. Only two studies included in the analysis [21, 22] evaluated TSA, which had smaller populations and fewer infection events. The microbiological and anatomical characteristics of shoulder arthroplasty may differ from those of lower-limb procedures; for example, *Cutibacterium acnes* predominates in TSA infections and may be less influenced by systemic nutritional status [39, 40]. Consequently, while the pooled data strongly support GNRI as a risk marker for SSI after hip and knee replacements, further well-designed, large-scale studies are needed to validate its relevance in shoulder arthroplasty.

An important consideration in interpreting the pooled results is the distinction between multivariable-adjusted and unadjusted effect estimates. Although the overall meta-analysis included a small number of unadjusted ORs when no adjusted models were available, the subgroup analysis indicated that the association between low GNRI and SSI risk was primarily driven by studies employing multivariable adjustment, which yielded consistent and statistically significant effects. In contrast, univariate analyses did not demonstrate a significant association. This divergence strongly suggests that crude associations are substantially influenced by confounding factors—such as comorbidity burden, metabolic status, frailty, and functional impairment—and that adequate confounding control materially alters the observed relationship. Therefore, the inclusion of unadjusted effects in the overall pooled analysis may have modestly attenuated the summary estimate toward the null, and the adjusted estimates should be regarded as the more informative basis for prognostic interpretation.

Although the exploratory analysis of PJI did not demonstrate a statistically significant relationship, the direction and magnitude of the association (OR > 2) were consistent with the results for SSI. This likely reflects an underpowered analysis due to the limited number of events and studies reporting PJI-specific data. Given that PJI represents a subset of SSI with more severe clinical consequences and longer latency, longer-term follow-up data may be required to fully capture its occurrence [41]. Although the numerical estimate suggested a directionally similar pattern to the SSI findings, only two studies (three datasets) contributed data, resulting in substantial imprecision and moderate heterogeneity. The synthesis regarding PJI is therefore exploratory and hypothesis-generating rather than confirmatory, and more high-quality studies are needed before making firm conclusions.

This meta-analysis possesses several strengths. It represents the most comprehensive and up-to-date synthesis of evidence on GNRI and infection risk after TJA, incorporating all available cohort data up to 2025. All included studies were longitudinal in design, ensuring a clear temporal relationship between preoperative nutritional assessment and postoperative outcomes. Multiple sensitivity and subgroup analyses were performed to confirm the robustness of findings across surgical types, joint locations, follow-up durations, and analytical models. The consistency of results across most subgroups, combined with the absence of significant publication bias, lends credibility to the conclusion that GNRI may serve as a predictor of postoperative SSI risk after TJA.

However, several limitations should be acknowledged. First, while regression-derived ORs were pooled as independent estimates, some datasets originated from multiple GNRI severity contrasts within the same cohort. These contrasts are not fully independent at the study-population level and may introduce within-study correlation, potentially leading to a slight overestimation of precision. Nonetheless, this is unlikely to materially affect the main interpretation, as the direction of association was consistent across GNRI categories, the pooled results were robust in sensitivity analyses, and between-study heterogeneity was substantial. This approach was adopted to retain clinically relevant information on malnutrition severity in the context of a limited evidence base and should be interpreted accordingly. Additionally, all included studies employed retrospective cohort designs, which are inherently susceptible to recall bias, incomplete data recording, and potential residual confounding [42]. The presence of significant statistical heterogeneity likely reflects differences in patient demographics, comorbidities, surgical techniques, prosthesis types, and institutional practices. Variability in surgeon experience, perioperative antibiotic protocols, and hospital case volume—known determinants of infection risk—were inconsistently reported and could not be uniformly adjusted for. The PI (0.73–3.47) highlights the variability expected across clinical settings. Formal meta-regression was not attempted due to the limited number of studies, which would yield statistically unreliable estimates. Instead, prespecified subgroup comparisons—such as joint type, primary vs revision surgery, and GNRI severity—provided the same contrasts that would be obtained through meta-regression on these categorical modifiers. However, the subgroup analyses should be interpreted with caution. With only seven studies available, these comparisons were exploratory in nature and primarily hypothesis-generating, and multiple testing may inflate the likelihood of chance findings. Although the observed patterns aligned with clinical expectations, they should not be regarded as definitive effect modifiers. Furthermore, most studies were conducted in the United States among elderly populations, limiting generalizability to other healthcare systems or younger patients. Due to the availability of only aggregated data, individual patient-level analyses could not be performed to adjust for overlapping or interaction effects among variables such as diabetes, obesity, or inflammatory markers. Confounding remains a significant limitation of this meta-analysis. Several clinical factors—such as diabetes mellitus, obesity, overall comorbidity burden, frailty or functional status, and inflammatory conditions—can influence both preoperative nutritional status and susceptibility to postoperative infection. Although most included studies reported multivariable models, the covariates used were not standardized, and key confounders were variably included across studies. Even among the adjusted analyses, residual confounding is likely, and the difference between univariate and multivariable estimates suggests that confounding may materially influence the observed association. Due to the high heterogeneity of adjustment sets, a sensitivity analysis restricted to uniformly ‘adequately adjusted’ studies was not feasible. Moreover, the observational nature of the included studies precludes causal inference, and the findings should be interpreted as indicative of an association rather than a direct cause-and-effect relationship. Finally, while Egger’s test (*P ═* 0.31) and the visually symmetric funnel plot suggested no clear evidence of small-study bias, these findings should be interpreted cautiously as the funnel plot included 13 datasets derived from only seven studies. Several datasets originated from different GNRI severity categories within the same cohort and thus do not represent fully independent studies. Additionally, although the direction of effect for PJI was similar to that for SSI, the evidence base was extremely limited, and mixing adjusted and unadjusted estimates did not account for the non-significance; rather, the analysis was underpowered. Future prospective studies with standardized adjustment and adequate sample sizes are needed to clarify this potential association.

Despite these limitations, the findings of this meta-analysis carry significant clinical implications. Nutritional screening using GNRI is simple, inexpensive, and feasible to perform preoperatively. Incorporating GNRI into preoperative risk stratification models may enhance patient selection, inform shared decision-making, and reduce postoperative complications. Future research should aim to validate GNRI-based risk thresholds across diverse populations, investigate the efficacy of nutritional optimization in randomized or prospective studies, and explore the integration of GNRI with other inflammatory or metabolic biomarkers to refine prediction models. However, it should be emphasized that while lower GNRI values are associated with higher infection risk, this relationship must not be interpreted as causal. All included studies were retrospective and subject to residual confounding; no available data demonstrate that improving GNRI or correcting malnutrition directly reduces SSI or PJI. Therefore, GNRI should be viewed as a risk stratification tool that may help identify patients who warrant closer perioperative attention rather than as a target for proven risk modification. Whether nutritional interventions—such as protein supplementation, multimodal prehabilitation, or correction of hypoalbuminemia—can reduce postoperative infection risk remains unanswered. Randomized trials or rigorously designed prospective studies are required before GNRI-guided nutritional optimization can be recommended as standard practice. Current evidence supports risk prediction, not risk modification.

## Conclusion

In conclusion, this meta-analysis suggests that preoperative malnutrition, as reflected by a low GNRI, may be associated with an increased risk of SSI after TJA. Although the pattern appears directionally consistent across primary and revision procedures and is more pronounced with greater nutritional impairment, the certainty of evidence remains limited due to the retrospective design of available studies and substantial heterogeneity. GNRI may serve as a practical preliminary indicator for identifying patients who could benefit from closer perioperative attention, but its clinical use should be viewed as conditional and requires confirmation in high-quality prospective studies. Nutritional assessment and optimization before TJA remain important components of holistic perioperative care, but any GNRI-guided strategies should be considered exploratory.

## Supplemental data

**Supplemental file 1.** Detailed search strategy for each database

PubMed

((“Geriatric Nutritional Risk Index”[Mesh] OR “geriatric nutritional risk index”[tiab] OR GNRI[tiab] OR malnutrition[Mesh] OR malnutrition[tiab] OR “nutritional indices”[tiab] OR “nutritional index”[tiab]) AND (“Arthroplasty, Replacement, Hip”[Mesh] OR “Arthroplasty, Replacement, Knee”[Mesh] OR “Arthroplasty, Replacement, Shoulder”[Mesh] OR “total joint arthroplasty”[tiab] OR “total joint replacement”[tiab] OR “total hip arthroplasty”[tiab] OR “total knee arthroplasty”[tiab] OR “total shoulder arthroplasty”[tiab] OR “total hip replacement”[tiab] OR “total knee replacement”[tiab] OR “total shoulder replacement”[tiab] OR “joint replacement”[tiab] OR THA[tiab] OR TKA[tiab] OR TJA[tiab]))

**Embase**

(‘geriatric nutritional risk index’/exp OR ‘geriatric nutritional risk index’:ti,ab OR GNRI:ti,ab OR ‘malnutrition’/exp OR malnutrition:ti,ab OR ‘nutritional index’/exp OR ‘nutritional indices’:ti,ab) AND (‘total joint arthroplasty’/exp OR ‘arthroplasty’/exp OR ‘total joint replacement’:ti,ab OR ‘total hip arthroplasty’:ti,ab OR ‘total knee arthroplasty’:ti,ab OR ‘total shoulder arthroplasty’:ti,ab OR ‘total hip replacement’:ti,ab OR ‘total knee replacement’:ti,ab OR ‘total shoulder replacement’:ti,ab OR ‘joint replacement’:ti,ab OR THA:ti,ab OR TKA:ti,ab OR TJA:ti,ab)

**Web of Science**

TS ═ ((“geriatric nutritional risk index” OR GNRI OR malnutrition OR “nutritional indices” OR “nutritional index”) AND (“total joint arthroplasty” OR “total joint replacement” OR “total hip arthroplasty” OR “total knee arthroplasty” OR “total shoulder arthroplasty” OR “total hip replacement” OR “total knee replacement” OR “total shoulder replacement” OR “joint replacement” OR THA OR TKA OR TJA))

**Table S1:** Definitions of SSI/PJI in each study

**Study**	**SSI/PJI definitions**	**Follow-up window (days)**
Fang 2022	SSI: Includes both superficial and deep incisional infections as defined by standard surgical outcomes criteria.	30
Oakley 2023	SSI: Includes both superficial and deep incisional infections as defined by standard surgical outcomes criteria. PJI: Included as a major complication within 90 days.	90
Lung 2024	SSI: Includes both superficial and deep incisional infections as defined by standard surgical outcomes criteria.	30
Liu 2024a	SSI: Includes both superficial and deep incisional infections as defined by standard surgical outcomes criteria.	30
Liu 2024b	SSI: Includes both superficial and deep incisional infections as defined by standard surgical outcomes criteria.	30
Liu 2025	SSI: Includes both superficial and deep incisional infections as defined by standard surgical outcomes criteria.	30
Hansen 2025	SSI: Includes both superficial and deep incisional infections as defined by standard surgical outcomes criteria. PJI: Defined using the 2018 International Consensus Meeting criteria	90

Abbreviations: SSI: Surgical site infection; PJI: Periprosthetic joint infection; ICM: International Consensus Meeting.

## Data Availability

All data generated or analyzed during this study are included in this published article.
